# The PCNA Interaction Protein Box Sequence in Rad54 Is an Integral Part of Its ATPase Domain and Is Required for Efficient DNA Repair and Recombination

**DOI:** 10.1371/journal.pone.0082630

**Published:** 2013-12-20

**Authors:** Rebecca C. Burgess, Marek Sebesta, Alexandra Sisakova, Victoria P. Marini, Michael Lisby, Jiri Damborsky, Hannah Klein, Rodney Rothstein, Lumir Krejci

**Affiliations:** 1 Department of Genetics & Development, Columbia University Medical Center, New York, New York, United States of America; 2 National Centre for Biomolecular Research, Masaryk University, Brno, Czech Republic; 3 Department of Biology, Faculty of Medicine, Masaryk University, Brno, Czech Republic; 4 International Clinical Research Centre, Centre for Biomolecular and Cellular Engineering, Saint Anne's University Hospital, Brno, Czech Republic; 5 Department of Molecular Biology, University of Copenhagen, Copenhagen N, Denmark; 6 Department of Experimental Biology, Faculty of Science, Masaryk University, Brno, Czech Republic; 7 Department of Biochemistry and Molecular Pharmacology, New York University School of Medicine, New York, New York, United States of America; Tulane University Health Sciences Center, United States of America

## Abstract

Rad54 is an ATP-driven translocase involved in the genome maintenance pathway of homologous recombination (HR). Although its activity has been implicated in several steps of HR, its exact role(s) at each step are still not fully understood. We have identified a new interaction between Rad54 and the replicative DNA clamp, proliferating cell nuclear antigen (PCNA). This interaction was only mildly weakened by the mutation of two key hydrophobic residues in the highly-conserved PCNA interaction motif (PIP-box) of Rad54 (Rad54-AA). Intriguingly, the *rad54-AA* mutant cells displayed sensitivity to DNA damage and showed HR defects similar to the null mutant, despite retaining its ability to interact with HR proteins and to be recruited to HR foci *in vivo*. We therefore surmised that the PCNA interaction might be impaired *in vivo* and was unable to promote repair synthesis during HR. Indeed, the Rad54-AA mutant was defective in primer extension at the *MAT* locus as well as *in vitro*, but additional biochemical analysis revealed that this mutant also had diminished ATPase activity and an inability to promote D-loop formation. Further mutational analysis of the putative PIP-box uncovered that other phenotypically relevant mutants in this domain also resulted in a loss of ATPase activity. Therefore, we have found that although Rad54 interacts with PCNA, the PIP-box motif likely plays only a minor role in stabilizing the PCNA interaction, and rather, this conserved domain is probably an extension of the ATPase domain III.

## Introduction

Homologous recombination (HR) is a conserved and vital repair mechanism used for a spectrum of genome maintenance processes, such as repair of DNA lesions, restart of replication fork progression, telomere lengthening, and proper chromosome segregation during meiosis (reviewed in [1], [2]). In budding yeast, the main steps of HR are carried out by a highly-conserved set of proteins encoded by the *RAD52* epistasis group (reviewed in [3], [4]). The process of HR is initiated by the nucleolytic processing of a double-strand DNA break (DSB) into 3′ single-stranded DNA (ssDNA) tails, which are rapidly coated by the single-strand binding protein, RPA. With the help of the Rad52 protein, RPA is then replaced by Rad51 to form a nucleoprotein filament, which mediates the search for homologous donor DNA. Invasion of the Rad51-coated DNA end into a homologous donor template (termed synapsis) produces heteroduplex DNA and displaces the complementary strand to produce a structure known as a displacement loop (D-loop). The invading end then serves as a priming site for DNA synthesis to replace the lost and/or damaged sequence. Once repair synthesis is complete, the DNA intermediates are resolved and bound proteins removed, allowing the cell to resume normal cell cycle progression and growth.

Rad54, a member of the Swi/Snf2 protein family, has been reported to act in multiple steps of HR from pre-synaptic to post-synaptic phases (reviewed in [5], [6], [7]). Rad54 contains the classical seven motifs of the SF2 superfamily of helicases/translocases, which mark proteins that translocate on DNA in an ATP hydrolysis-driven fashion. Despite its SF2 membership, Rad54 likely has both ATPase-dependent and -independent roles in HR [8], [9]. The pre-synaptic stabilization of Rad51 filaments by Rad54 is ATPase-independent [10] and is likely mediated by Rad51-Rad54 physical interactions [11], [12], [13]. During synapsis, DNA strand invasion and subsequent D-loop formation is also facilitated by the action of the Rad54 protein, possibly due to its dsDNA-dependent translocase and/or chromatin remodeling activities that allow sampling and accessibility of donor DNA sequences [9], [14], [15], [16], [17], [18], [19]. In addition, Rad54 has been reported to act after the Rad51 invasion step of HR [20]. This protein exhibits several biochemical activities that may also be used in its post-synaptic role: ATP-dependent branch migration activity, as well as translocation on dsDNA and removal of Rad51 [21], [22], [23], [24]. Displacement of Rad51 by Rad54 is postulated to free the 3′OH of the invading DNA end to allow priming of repair synthesis [20], followed by primer extension via the proliferating cell nuclear antigen (PCNA)-dependent DNA polymerase delta [20], [25], [26], [27], [28].

Finally, Rad54 has also been implicated in the resolution of replication and recombination intermediates [29], [30]. Genetic studies on *RAD54* mutants also support the notion for its role in post-synaptic steps of the HR pathway: *rad54Δ* mutants are synthetic lethal with *srs2Δ*, since in the absence of Srs2, lesions are increasingly channeled into HR which cannot be efficiently completed without Rad54 [31], [32], [33]. The many potential roles of Rad54 and how these are choreographed throughout the HR process remains unclear.

In this work, we probed the function of a conserved PCNA interaction motif within Rad54, the PCNA interacting protein box (PIP-box) [34]. PCNA is a homotrimeric sliding clamp that functions as a processivity factor to various DNA polymerases and interacts with numerous proteins often via a PIP-box motif, although there are some notable exceptions involving interactions with post-translational modifications of PCNA [35], as well as a recently described AlkB homologue 2 PCNA interaction motif (APIM) [36]. PIP-domains form a hydrophobic plug that binds to PCNA through a hydrophobic pocket in the interdomain-connecting loop [37], [38]. It is likely that many PIP-containing proteins compete for the hydrophobic pocket of PCNA. However, since PCNA is a homotrimer, it is possible that PCNA binds multiple binding partners simultaneously, perhaps to facilitate enzyme exchange such as in the ‘tool belt’ model of translesion repair polymerase exchange [39].

PCNA has long been implicated in repair processes, as multiple PCNA mutants have been described that result in increased DNA damage sensitivity [40]. Importantly, in the *pcna-79* mutant that disrupts the interdomain connecting loop (IDCL) to which PIP-containing proteins bind, cells show increased sensitivity to the DNA damaging agents methyl methane sulfonate (MMS), hydroxyurea (HU) and ultraviolet (UV) light [41]. It has been postulated that PCNA scans the genome for damage and might recruit repair proteins and auxiliary components such as chromatin assembly factors to break sites [42], [43]. The PIP domain in Rad54 is very well conserved in flies, worms, mice and humans, suggesting that this domain is important for HR function in higher eukaryotes.

Here we wanted to decipher the role of Rad54-PCNA interactions, in the hopes that it might provide clues to the multiple roles of the Rad54 protein in HR, particularly in assembly of the DNA repair synthesis machinery. Our results show that Rad54 can indeed bind directly to PCNA, but surprisingly, the mutation of two critical hydrophobic residues in the Rad54 PIP-box just slightly weakens this interaction. Arguing for the domain's importance for *in vivo* function, this mutant showed significant defects in recombination assays and in the primer extension step of DNA repair synthesis. However, the HR phenotype of the PIP-box mutant seems to be attributable to defects in Rad54 ATPase activity, rendering its branch migration, and D-loop functions ineffective. In fact, other mutations in the canonical PIP-box that showed evidence for recombination defects *in vivo* were also ATPase-defective. Overall, our data suggests that the putative PIP-box motif within Rad54 might be an integral part of its ATPase domain and is not essential for PCNA interaction.

## Results and Discussion

### Rad54 directly interacts with PCNA

**Figure 1:**
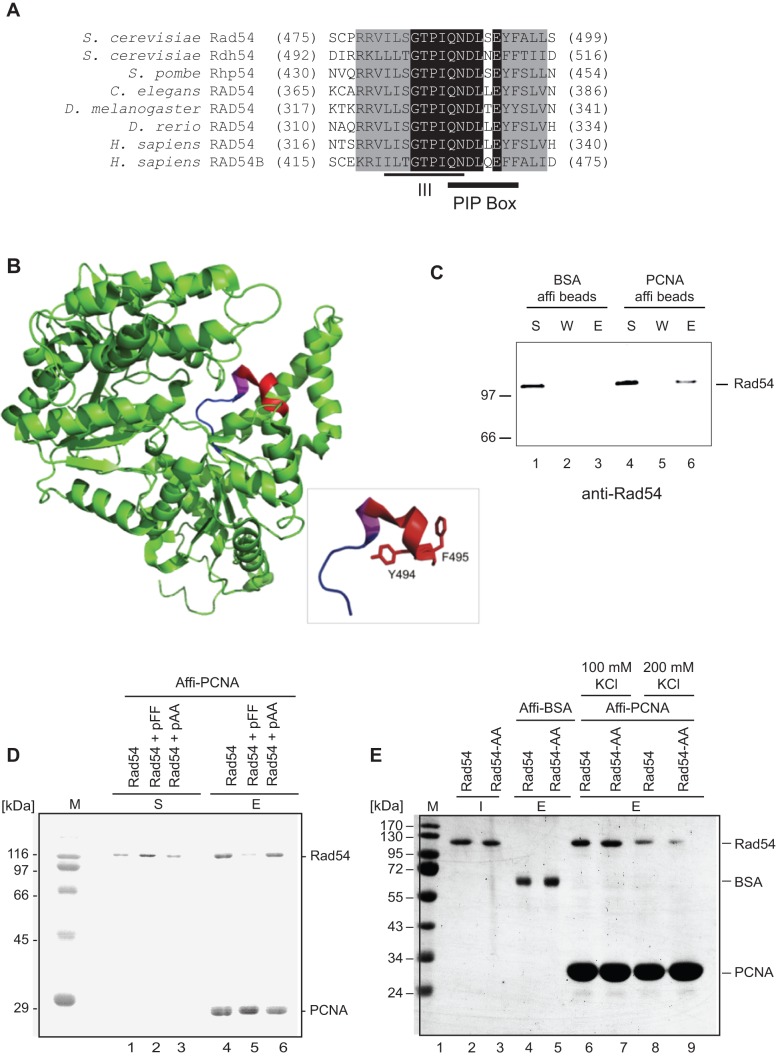
The Rad54 family contains a conserved PCNA interaction motif, and yeast Rad54 directly interacts with PCNA. **A. Alignment of the Rad54 family of proteins.** The conservation of the amino acid sequence around motif III in Rad54 family members from budding yeast to human is depicted. Amino acids shaded in black are identical between the sequences, while grayed amino acids retain highly similar side chains. The location of the PCNA interaction protein box (PIP-box) is shown below the alignment. The two C-terminal highly-conserved aromatic residues were changed to alanines by site-directed mutagenesis to create the Rad54-AA (Y494A, F495A) mutant protein. **B. Section of the Rad54 structure containing the PIP box and ATPase domains.** The tertiary structure of the protein Rad54 from *D. rerio* (Zebrafish (PDB ID 1Z3I)) is represented in the ribbon diagram. Motif III (317-ISGTPIQN-324, corresponding to amino acids 481–489 in *S. cerevisiae* Rad54) is shown in blue, the PIP-box motif (323-QNDLLEYF-330, corresponding to amino acids 488–495 in *S. c*. Rad54) is shown in red, while overlapping residues assigned to both motifs (323-QN-324) are shown in magenta. The detailed view (inset) shows these two motifs and mutated residues Y329 (494) and F330 (495) in stick representation. **C. Rad54 interacts with PCNA in vitro.** Rad54 protein was mixed with either control beads (affi-BSA, lanes 1–3) or with PCNA immobilized on affi-beads (lanes 4–6). After incubation for 30 min at 4°C with occasional mixing, the reaction mixtures were centrifuged and the supernatant was separated from the beads. Next, the beads were washed and the proteins were eluted by SDS-PAGE loading dye. Supernatant (S), wash (W) and eluate (E) fractions were separated on a 12% SDS-PAGE gel, followed by blotting and detection with α-Rad54 antibodies. **D. Oligopeptides derived from the PIP-box outcompete PCNA for interaction with Rad54.** In the pull-down experiments shown, Rad54 was mixed with immobilized PCNA in the presence of an oligopeptide derived from the Rad54 PIP box (pFF; lanes 2, 5) or its mutated version (pAA; lanes 3, 6). Lanes 1, 4 represent control experiment where no peptide was added. The reactions were performed as described in C. Supernatant (S), and eluate (E) fractions were separated on a 12% SDS-PAGE gel, followed by Coomassie staining. **E. PCNA interaction with Rad54 and Rad54-AA.** In these pull-down experiments, Rad54 (lanes 6 and 8) or Rad54-AA (lanes 7 and 9) were mixed with PCNA immobilized on affi-beads, or with BSA affi-beads as a control (lanes 4, 5). The reactions were performed as described in C. Input (I) and eluate (E) fractions were separated on a 12% SDS-PAGE gel, followed by Coomassie staining.

Sequence analysis of the Rad54 family of proteins revealed the presence of a conserved PCNA interaction protein motif, or PIP-box, immediately adjacent to the central motif III of the Snf2 family ATPase domains (Fig. 1A and B). We confirmed that Rad54 directly interacts with PCNA using purified proteins in pull-down experiments (Fig. 1C). In addition, a peptide containing the Rad54 PIP-box domain was capable of competing with full-length Rad54 for interaction with PCNA (Fig. 1D), indicating that the Rad54 binding site is likely near the interdomain-connecting loop of PCNA.

It has been shown that two aromatic residues located within the PIP motif are essential for interaction with PCNA [44]. Therefore, we tested whether a mutant Rad54 protein in which the two highly conserved aromatic residues Y494 and F495 were changed to alanine (Rad54-AA) could bind to PCNA. Accordingly, a peptide derived from the Rad54-AA mutant PIP-box (pAA) was unable to compete with full-length Rad54 for interaction with PCNA, in contrast to the corresponding intact PIP-box peptide (pFF, Fig. 1D), indicating that the interaction between Rad54 and PCNA is mediated by the PIP-box and IDCL domains To determine whether the PIP-box in full-length Rad54 protein was responsible for the interaction with PCNA, we purified both wild type Rad54 and Rad54-AA mutant proteins in parallel. Rad54 wild type protein efficiently binds PCNA, albeit with lower affinity at 200 mM KCl than at 100 mM KCl. Surprisingly, Rad54-AA protein shows proficient binding to PCNA at 100 mM KCl, and only slightly diminished binding at 200 mM compared to the wild-type Rad54 (Fig. 1E), suggesting that this PIP-box is utilized for the interaction only to a small extent. In summary, our pull-down experiments suggest that Rad54 binds to the IDCL domain in PCNA, however, the putative PIP-box in Rad54 plays only a minor role in the interaction. Therefore, it is possible that Rad54 binds PCNA through an alternative, yet to be determined domain, such as the PIM (PCNA-interaction motif), which also binds to IDCL domain on PCNA [45], [46], or APIM (AlkB homologue 2 PCNA-interaction motif) [36].

Moreover, individual interactions between PCNA and Rad54 may be transient in nature and the PIP-domain may still play a role in stabilizing the overall Rad54-PCNA interaction, since reported PIP-box-PCNA interactions range from fairly weak to very strong [34]. The Rad54-PCNA interaction is on the weak side, unlike the strong Pol32 interaction, which interacts through both the PIP-domain and other domains [47]. On the other hand, the Rad54-Rad51 interaction is extremely strong, and is unimpeded by the PIP-box mutation in Rad54 (Fig. S1A in File S1). In fact, Rad51 can outcompete PCNA for interaction with Rad54 (Fig S1B in File S1), indicating that the PCNA binding is weaker than Rad51, and the two proteins overlap in their binding sites. Although many proteins interact with PCNA, Rad54 is the first protein that is also involved in promoting homologous recombination. It is therefore tempting to speculate that interaction with Rad54 could govern the progression of recombination from synaptic (Rad51) to post-synaptic (PCNA) modes via such a competition.

### The *rad54* PIP-box mutant has defects in resistance to DNA damaging agents and in homologous recombination

**Figure 2:**
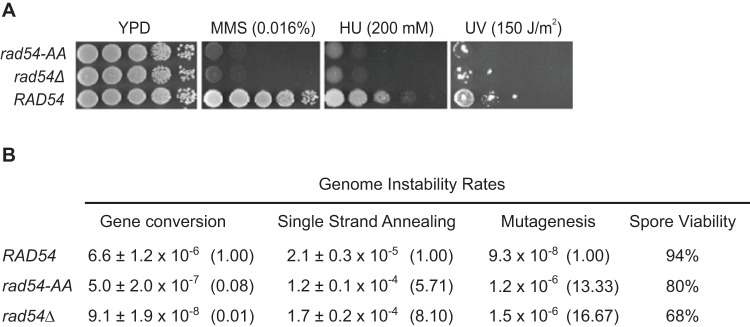
The Rad54 PIP-box mutant renders cells defective in homologous recombination and genome stability functions. **A. Cells with the *rad54-AA* mutation are as sensitive to DNA damaging agents as the null mutant.** Shown are 10-fold dilutions of cells spotted onto plates treated with methylmethane sulfonate (MMS), hydroxyurea (HU) or ultraviolet (UV) light, at the indicated doses. The spot assay was performed as described in the methods. **B.** **The** ***rad54*** **PIP-box mutant is defective in mitotic recombination and genome stability functions**. Results from wild type, *rad54*Δ and *rad54-AA* mutant strains in recombination assays are shown, and the fold change from the wild type is shown in parentheses. Gene conversion and deletion (single-strand annealing) event rates were determined by fluctuation tests with the *leu2-EcoRI::URA3-leu2-BstEII* reporter as described in the methods. The mean of the rates from three independent experiments are shown with standard deviations. Spontaneous mutagenesis rates in the *rad54-AA* mutant were determined two or three times by fluctuation tests, as described. Significance was determined using a t-test (p<0.05). For spore viability, diploids homozygous for *RAD54, rad54Δ*, or *rad54-AA* were sporulated and dissected, and the surviving spores quantified. At least 100 spores were analyzed for each strain.

The slight loss of Rad54-AA interaction with PCNA in near-physiological salt suggested it might have a more profound defect in cellular conditions. Therefore, we tested whether the mutant retained its biological function to repair spontaneous and induced DNA damage and promote homologous recombination *in vivo.* Null mutations in *RAD54* show sensitivity to a variety of DNA damaging agents including ionizing radiation [48], a decrease in gene conversion events (GC), and a concomitant increase in the rate of single-strand annealing events (SSA) [49], [50]. We found that the *rad54-AA* mutant is sensitive to MMS, HU and UV damage and its sensitivities were indistinguishable from the *rad54Δ* strain (Fig. 2A). Likewise, spore viability, a measure of successful meiotic recombination, was impaired in the *rad54-AA* mutant, although not quite as severe as in the *rad54Δ* mutant (Fig. 2B).

The effect of the *rad54-AA* mutant on the rate of spontaneous recombination was measured using a recombination cassette at the *LEU2* locus where two non-functional *leu2* alleles flank *URA3*. In this assay, gene conversion rates are measured by the number of events producing a functional *LEU2* gene, while rates of another Rad51-independent HR pathway, single-strand annealing (SSA), are determined by the number of events where the intervening *URA3* sequence is lost [51]. Gene conversion events, Leu^+^ Ura^+^ recombinants, were reduced 14-fold in the *rad54-AA* mutant, while deletion or single-strand annealing events were increased 6-fold (Fig. 2B). These recombination rates in the *rad54-AA* mutant are similar to that found in the deletion mutant. Lastly, we measured spontaneous mutagenesis at the *CAN1* locus and observed an increase of 13-fold, again a level similar to that in the *rad54Δ* strain (Fig. 2B). These data indicate that the *rad54-AA* strain is impaired for HR in the repair of spontaneous damage during replication and hence error-prone repair pathways are used instead. Taken together, we conclude that the Rad54 PIP-box is critical for its function in homologous recombination repair.

### Rad54 PIP-box mutant cells show defects in the post-synaptic stage of recombination

The role of PCNA as a replication clamp immediately suggested a function for this interaction in the repair synthesis step of recombination, so we tested whether this interaction affected later step in HR. Interestingly, we observed an increase in the number of spontaneous Rad52 recombination foci in the presence of the *rad54-AA* mutant, to levels similar to the *rad54Δ* (Fig. S2A in File S1). Increases in spontaneous Rad52 foci can result from different scenarios. Either the incidence of Rad52 foci increases during the cell cycle, or protein foci (occurring with the same frequency as wild type) last longer due to a defect in recombination or disassembly of repair complexes. To distinguish between these possibilities, we measured Rad52 focus incidence and duration using time-lapse microscopy. Interestingly, the average duration of a Rad52 focus was over 3 times longer in *rad54-AA* or *rad54Δ* cells compared to wild type cells, while the incidence remained unchanged (Fig. S2B in File S1). This result led us to hypothesize that the *rad54-AA* mutant had a defect in the completion of HR, since the foci were not disassembled promptly. A later role of Rad54 in recombination was proposed to be responsible for synthetic lethality when deleted together with the *SRS2* gene [52]. Fittingly, *rad54-AA* also shows synthetic lethality with *srs2Δ* (Fig. S2C in File S1) supporting the notion that the mutant could be defective in a post-synaptic HR step.

**Figure 3:**
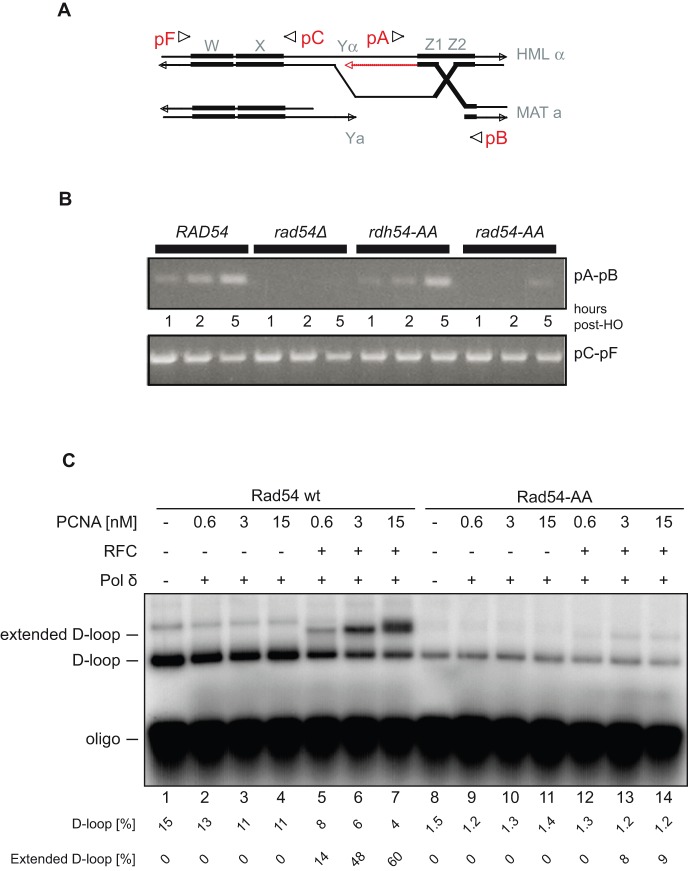
Rad54-AA is defective in strand invasion and primer extension activities. **A. Schematic of the *MAT* chromosomal locus used for the examination of DNA repair synthesis.** Arrows depict the direction of primers used for detection of primer extension intermediates by PCR. **B.** **The primer extension step of recombination is compromised in the** ***rad54*** **PIP-box mutant.** The top panel shows the formation pA-pB product, which results from minimal DNA synthesis from the invading strand. Samples were taken at 1, 2 or 5 h after HO endonuclease cutting. The bottom panel shows pC-pF control product. **C. Rad54-AA is defective in DNA repair synthesis** ***in vitro.*** Rad51 and DNA substrates were pre-formed into nucleoprotein filaments as described, then either Rad54 wild type (wt, lanes 1–7) or Rad54-AA (lanes 8–14) was incorporated and D-loop formation was initiated. DNA synthesis reactions were then performed using Polymerase δ (15 nM), and increasing concentrations (2.5, 5, 10, 20 nM) of the PCNA clamp, with or without the PCNA clamp loader, RFC (10 nM), in the presence of RPA (666 nM). The reactions were monitored using labeled α-[^32^P]-dATP, and percentage of each reaction product shown below.

We then tested whether the *rad54-AA* mutant was affected in its ability to carry out the repair synthesis steps of recombination. To this end, we used an assay developed by the Haber lab to detect primer extension intermediates produced by DNA synthesis following strand invasion from an HO-induced DSB at the *MAT* locus in living cells [33]. In this reaction, Rad54 is required for production of the primer extension intermediate, but not for earlier steps of Rad51 binding to the DSB, or for synapsis with the homologous donor template ([33]; Fig. 3A). We find that the *rad54-AA* mutation renders cells defective in formation of primer extension intermediates (Fig. 3B). This was also confirmed by our *in vitro* reconstituted DNA repair extension system ([27]; Fig. 3C). However, the formation of primer extension intermediates was not completely blocked and residual product (about 20% of wild type levels) is still formed in the presence of *rad54-AA* (Fig. 3B). This defect is specific to the Rad54 PIP-box mutant, since the corresponding mutation in the Rad54 paralog Rdh54, did not affect DNA repair synthesis in this assay (Fig. 3B). This result suggests that the PIP-box domain of Rad54 is critical for its HR functions *in vivo*, particularly for late recombination steps such as repair synthesis.

### The Rad54-AA mutant protein is deficient in most of its biochemical activities

Since the *in vivo* interaction with Rad51 was unaffected by the *rad54* PIP-box mutation, but showed a profound recombination defect (Fig. S1A in File S1 and 2B, respectively), we tested whether this defect might lie in the inability of the Rad54-AA protein to localize to Rad52 recombination centers. Therefore, we fused *rad54-AA* at its endogenous chromosomal locus to the YFP reporter gene and examined its localization using fluorescence microscopy. As shown in Fig. S2D in File S1, the Rad54-AA-YFP protein forms foci and colocalizes with Rad52 foci about 70% of the time, which is indistinguishable from the 75% of wild type Rad54 foci that colocalize with Rad52 foci. This indicated that the Rad54-AA mutant protein is fully capable of being recruited to sites of HR in the cell. Altogether, these data suggest that the Rad54 PIP-box mutation does not affect the Rad51-Rad54 interaction or recruitment to recombination centers.

However, since the PIP-box mutant shows a recombination phenotype similar to that of Rad54 deletion mutant, we checked the effect of this mutation on other Rad54-mediated biochemical properties, particularly ones required for later steps in HR. Therefore, we compared the D-loop formation activities of wild type and the Rad54-AA protein. While we found a significant defect in D-loop formation when performing the *in vitro* DNA extension assay (Fig. 3C), we confirmed this observation by testing D-loop formation with a range of Rad54 protein concentrations, and found it defective in all tested conditions (Fig. S3 in File S1). These results indicate that the Rad54-AA protein, while retaining its interaction with Rad51, does not promote homologous pairing and subsequent extension.

**Figure 4:**
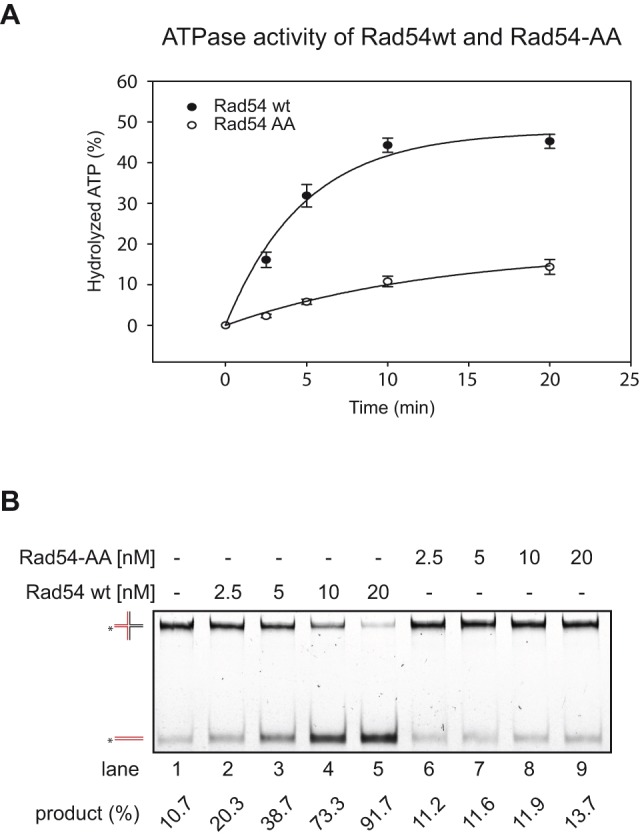
The rad54-AA mutant protein is deficient in most of its biochemical activities. **A. Rad54-AA has lower ATPase activity compared to wild type Rad54.** Rad54-AA and Rad54 wt (75 nM, each), respectively, were mixed with dsDNA and α-[^32^P]-labeled ATP. At indicated times, samples were withdrawn and analyzed by thin-layer chromatography. Error bars represent standard error produced by 3 experiments. **B. Rad54-AA does not branch migrate mobile Holliday junctions.** DNA substrate was incubated with increasing concentrations (2.5, 5, 10, 20 nM) of Rad54 wt (lanes 2–5) or Rad54-AA (lanes 6–9), respectively, in the presence of ATP. Lane 1 shows the no protein control reaction.

The failure to promote D-loop formation and DNA strand extension despite an intact Rad51 interaction is reminiscent of the ATPase defective Rad54 protein [53]. For this reason, we tested the ATP hydrolysis of the Rad54-AA mutant and observed dramatic reduction of ATPase activity compared to wild type protein (Fig. 4A). Since Rad54 protein also requires ATP hydrolysis for branch migration [24], we tested both proteins for this activity. As shown in Fig. 4B, again the PIP-box mutant was defective in branch migration of Holliday junctions at all concentrations tested. Taken together, these data indicate that the PIP-box region of Rad54 is an extension of the ATPase domain III of the conserved helicase motifs within the Swi2/Snf2 family of translocases (Fig. 1A).

### DNA binding and oligomerization of Rad54-AA protein

**Figure 5:**
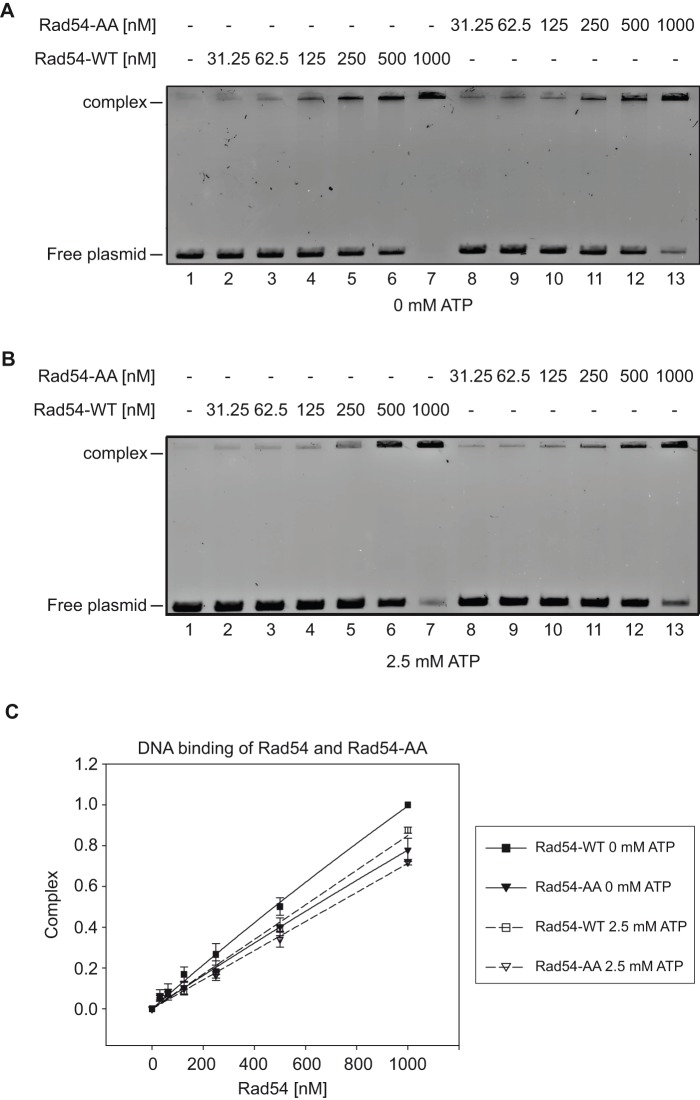
Rad54-AA cannot bind dsDNA as efficiently as wild type Rad54. **A. Rad54-AA performs less well than the wild type in a DNA binding assay in the absence of ATP.** Purified *S. cerevisiae* Rad54 and Rad54-AA (31.25, 62.5, 125, 250, 500 or 1000 nM) were incubated for 10 min with linearized pBluescript plasmid to assess DNA binding. Prior to gel electrophoresis, the proteins were cross-linked to DNA with 0.1% glutaraldehyde. After the addition of gel loading buffer, the reaction mixtures were resolved in a 0.8% agarose gel in TAE buffer and stained with Midori Green DNA stain. **B. Rad54-AA performs less well than the wild type in a DNA binding assay in the presence of ATP.** DNA binding assay as performed exactly as in A, except for the addition of 2.5 mM ATP, and an ATP-regenerating system to the reaction. **C. Quantification of the DNA binding reactions** shown in A and B. Error bars represent the standard error from three independent trials.

Since the ATP hydrolysis activity of Rad54 has been shown to be stimulated by dsDNA binding [13], we wished to test whether the ATPase defect of the Rad54-AA mutant was linked to an inability of this mutant to efficiently bind dsDNA. When using double-stranded plasmid DNA we found that the Rad54-AA mutant bound DNA slightly less efficiently than the wild type whether in the absence or presence of ATP (Fig. 5A and 5B, respectively). However, when we performed the assay using a short 49-mer dsDNA as substrate, we observed no difference between the wild-type and Rad54-AA proteins (Fig. S4 in File S1). Furthermore, an additional PIP-box mutant defective in ATPase activity (Rad54-L491Q (Rad54-L/Q)) showed similar DNA binding compared to wild type protein (Fig. S5 in File S1). Taken together, these results indicate that the PIP-box sequence of Rad54 plays only a minor role, if any, in the binding of dsDNA and is more likely to be directly required for the ATPase function of the protein.

### Mutational analysis of the PIP-box domain

Additional mutations to the *RAD54* PIP-box consensus sequence were generated to study the effect of amino acid changes in the hopes that they might differentially affect PCNA binding and/or ATPase activity, to separate these activities and study the PCNA interaction of Rad54 in isolation (Fig S6A in File S1). Substitutions of other key conserved residues, such as the L491Q, and Q488A mutants also drastically reduced ATPase and D-loop formation activities of the Rad54 protein, whilethe F495H (Rad54-F/H) substitution was the only mutation that retained these activities (Fig. S6B and C in File S1). The *rad54-F/H* mutant exhibited wild type resistance to MMS and a level of spontaneous Rad52 foci that was indicative of proficient/timely completion of HR (Fig. S6D in File S1). Since there was no appreciable defect in several *in vivo* homologous recombination and DNA repair assays (Fig. S6D in File S1 and data not shown), these results suggest that this mutant is not deficient for noteworthy functional interactions.

PCNA is involved in myriad interactions during multiple facets of DNA metabolism, including nearly all repair processes, and now we include an obligate homologous recombination protein to the list of interactors. How this interaction affects Rad54 function is unclear at the present, given the inability to isolate a separation-of-function mutant in which the PCNA interaction is abrogated while preserving ATPase activity. Although the PIP-box motif of the Rad54 family is a clear fit to the consensus sequence, it is probable that this domain is an extension of motif III of the ATPase domains, since several different mutations in this domain substantially affect this critical biochemical activity (Fig. 4, and S3 in File S1). A complementary study performed independently in the Heyer laboratory came to the same set of conclusions (see co-submitted ms.). Given the ability of the Rad54-AA mutant to only slightly reduce binding to PCNA under most biochemical conditions, Rad54 likely interacts with PCNA via multiple domains. Thus, extensive further mutational studies of Rad54 will be necessary to disrupt the cognate domains for dissecting the functional relevance of the Rad54-PCNA interaction. How, and if this interaction is linked to the inhibition of HR at replication forks by PCNA^SUMO^ interaction with Srs2 [54], [55], [56] is an important question for future study. Since the Rad54 sequence is highly conserved in *C. elegans*, *D. melanogaster*, as well as in mammalian cells, the PCNA interaction is likely also conserved, possibly for the orchestration and timely completion of HR, and the maintenance of genome integrity.

## Materials and Methods

### Yeast strains and plasmids

Strains used in this study are listed in Table S1. Standard yeast genetic techniques and growth conditions were used for creating and propagating strains [57]. Strains were grown at 30°C unless indicated otherwise. The *RAD54* and *RDH54* PIP-box mutations were produced in the *RAD5* derivative W303 yeast background [58], [59] by a cloning-free allele replacement method [60]. Primer sequences are available upon request; all replacements were confirmed by DNA sequencing.

Plasmid pRS316 with *rad54-Y494A F495A* was digested with *Avr*II and *Afl*II to release a fragment containing the *rad54-Y494A F495A* mutations. This fragment was used to replace the corresponding wild type fragment of *RAD54* inserted into pRS306. Subcloning of the *rad54-Y494A F495A* fragment was verified by the presence of a *Not*I site, which marks the *rad54-Y494A F495A* mutations and also by sequencing. The plasmid pRS306-*rad54-Y494A F495A* was digested with *Hin*dIII and the linear fragment was used to transform a wild type strain. After confirming the integration, the strain was passaged on medium containing 5-fluoro-orotic acid (5-FOA) to select for strains that had lost one copy of *RAD54*. Strains with *rad54-Y494A F495A* were confirmed by MMS sensitivity and DNA sequencing.

Plasmids for expression of Rad54 mutant versions (Rad54-AA, Rad54 Q488A (Rad54 Q/A), Rad54 L491Q (Rad54 L/Q), Rad54 F495H (Rad54 F/H) and Rad54 LF491,495QH (Rad54 LF/QH)) protein were generated by site-directed mutagenesis of a vector carrying wild type Rad54 [16].

### Recombination and genome stability assays

Gene conversion (Leu^+^ Ura^+^ segregants) and single-strand annealing (Ura^−^ segregants on 5-FOA medium) were performed using the *leu2-EcoRI::URA3-leu2-BstEII* reporter. Fluctuation tests were performed as described [46] using nine colonies for each test and performing the tests on three spore segregants for each genotype. Chromosome loss and mitotic recombination assays in diploids were performed as described [31] using fresh zygotes for each genotype. Nine zygotes were used for each test, and three tests were performed for each genotype. Mutation rates were calculated for canavanine resistant segregants as described [31]. Synthetic lethality was determined by mating a *rad54-AA* strain to an *srs2Δ* strain of opposite mating type. Diploids heterozygous for the *SRS2* and *RAD54* loci were then sporulated and dissected using standard techniques. Viable spores were scored for the *srs2Δ* genotype using histidine prototrophy conferred by the *HIS3* replacement of *SRS2*, and for *RAD54* using colony PCR, followed by digestion of the product with the *Not*I restriction enzyme, which specifically cleaves the *rad54-AA* mutation site.

Spore viability was determined by sporulation and dissection of fresh diploids homozygous for *RAD54, rad54-AA* or *rad54Δ*. Number of viable spores after 3 days growth on YPD were then counted and expressed as a percentage of the total number of spores dissected.

### Spot assay

Sensitivities to MMS, HU and UV were performed as described elsewhere [61].Live cell fluorescent microscopy of recombination proteins.

### Live cell fluorescent microscopy of recombination proteins

Yeast cells were prepared for microscopy as previously described and imaged on a Leica DM550B microscope described therein [62]. Volocity software (Improvision) was used to capture 11 Z-planes through the cells at 0.3 µm distances for focus frequency analyses. For Rad52 focus duration analyses, individual foci were followed over 5 minute intervals using time-lapse microscopy as described previously [63].

### Purification of Rad54 and its mutant forms

The expression and purification of Rad54, Rad54-AA, Rad 54 Q/A, Rad54 L/Q, Rad54 F/H and Rad54 LF/QH mutants were carried out as previously described [29].

### Purification of other proteins

Rad51, RPA, Polymerase δ, RFC and PCNA were purified as described previously [27].

### Binding of Rad54 to PCNA Affi-beads

Affi-gel 15 beads containing PCNA (Affi-PCNA; 5 mg/ml) or bovine serum albumin (Affi-BSA, 12 mg/ml) were prepared as described previously [64]. Purified Rad54 and Rad54-AA (3 µg of each), was mixed with 5 µl of Affi-PCNA or Affi-BSA in 30 µl of buffer K (10 mM Na_2_HPO_4_, 1.8 mM KH_2_PO_4_ pH 7.4 and either 100 mM or 200 mM KCl) for 30 min on ice. The beads were washed twice with 150 µl of the same buffer before being treated with 25 µl of 2% sodium dodecyl sulfate (SDS) to elute bound protein. The input (I), a supernatant containing unbound proteins (S), and the SDS eluate (E), were analyzed by 12% SDS polyacrylamide gel electrophoresis (PAGE) and staining with Coomassie Blue. The peptide competition assays were done in the presence of either pFF (VILSGTPI*QNDLSEYF*ALLSFSNP) or pAA (VILSGTPI*QNDLSEAA*ALLSFSNP) peptides derived from Rad54 or Rad54-AA sequence, respectively. The Rad51 competition was performed as described above. One reaction containing 4 µg of Rad51 was pre-incubated with Rad54 (3 µg) before applying the mixture on the Affi-PCNA beads. In the other reaction, 4 or 15 µg of Rad51 protein was included to the Rad54 and PCNA complex pre-assembled on Affi-PCNA beads, followed by washing and SDS elution as described above.

### Affinity pull-down

Purified Rad51 (3 µg) was incubated with Rad54 or Rad54-AA (3 µg each) in 30 µl of buffer T [20 mM Tris-HCl, pH 7.5, 150 mM KCl, 1 mM dithiothreitol (DTT), 0.5 mM EDTA, and 0.01% NP40] for 30 min at 4°C. The reactions were mixed with 15 µl Ni-NTA Agarose (Novagen) at 4°C for 30 min. After washing the beads twice with 150 µl of buffer T containing 150 mM KCl, the bound proteins were eluted with 30 µl of 5% SDS. The supernatant (S), wash (W) and SDS eluate (E) fractions (10 µl each), were analyzed on 12% SDS-PAGE.

### DNA substrates

Oligonucleotides were purchased from VBC Biotech and the sequences are shown in Table S2. All substrates were prepared by mixing an equimolar amount of the constituent oligonucleotides in the hybridization buffer as described in [29].

### ATPase assay

Rad54 and its mutant forms (75 nM each) were incubated at 30°C with pBluescript dsDNA (10 µM nucleotides), 1 mM ATP and 4 nCi/µl of [γ-^32^P] ATP at 30°C in buffer AA (30 mM Tris pH 7.5, 0.5 mM DTT, 0.1 mg/ml BSA, 0.9 mM MgCl_2_). Aliquots were withdrawn at 0, 2.5, 5, 10 and 20 min after the incorporation of Rad54 or its mutants. The reaction was stopped by adding SDS to 1% and reaction products were separated by thin layer chromatography on cellulose plates. These were analyzed by phosphorimaging using a scanner FLA-9000 Starion (Fujifilm) and the amount of labeled phosphate released during ATP hydrolysis was quantified with MultiGauge software (Fuji).

### Branch migration assay

Fluorescently labeled DNA substrate (6 nM) was incubated at 30°C with the indicated quantities of Rad54 or Rad54-AA in buffer D (25 mM Tris pH 7.5, 1 mM DTT, 0.1 mg/ml BSA, 50 mM KCl, 7.5 mM creatine phosphate, 11.25 µg/ml creatine kinase, 2.5 mM MgCl_2_ and 2.5 mM ATP) for 15 min. The reaction was stopped by the addition of SDS to 0.2% and proteinase K to 0.5 mg/ml followed by incubation at 30°C for 3 min. After adding loading buffer to the samples the reaction products were resolved by electrophoresis on a 10% native polyacrylamide gel in 1xTBE buffer. Gels were scanned using the image scanner FLA-9000 Starion imager (Fuji) and quantified by MultiGauge software (Fuji).

### DNA mobility shift assay

Purified Rad54 and Rad54-AA (31.25, 62.5, 125, 250, 500 or 1000 nM) were incubated with linearized pBluescript plasmid (30 µM as nucleotides) at 30°C in 10 µl of buffer D (40 mM Tris-HCl, pH 7.8, 50 mM KCl, 1 mM DTT, and 100 µg/ml BSA) for 10 min. Where indicated, 2.5 mM ATP, 3.5 mM MgCl_2_ and an ATP-regenerating system (10 µg/ml creatine phosphokinase and 20 mM creatine phosphate) were present in the reaction. Prior to gel electrophoresis, proteins were cross-linked to the DNA by addition of glutaraldehyde to a final concentration of 0.1%, followed by incubation at 30°C. After the addition of gel loading buffer, the reaction mixtures were resolved in 0.8% agarose gel in TAE buffer at 4°C. After electrophoresis, the gel was stained with Midori Green DNA stain (Nippon Genetics). The DNA species were visualized and quantified in the Fuji FLA 9000 Starion imager (Fuji) with the Multi Gauge software (Fuji).

### D-loop reaction and extension assay

The reactions were carried out essentially as described in Krejci et al. [64]. Briefly, the fluorescently labeled oligonucleotide D1 (3 µM nucleotides) was incubated with Rad51 (1 µM) for 5 min at 37°C to assemble Rad51-ssDNA nucleoprotein filaments. After incorporation of Rad54 or its mutant forms (75, 150 and 300 nM, respectively) the reactions were incubated for 3 min incubation at 23°C. D-loop formation was initiated by the addition of pBluescript replicative form I DNA (50 µM base pairs). The reaction mixtures were incubated at 30°C for 5 min, deproteinized by treatment with SDS (0.5%) and proteinase K (0.5 mg/ml) at 37°C for 5 min, and then run in a 1% agarose gel in TAE buffer. The gel was subjected to fluorescent imaging analysis in an FLA-9000 Starion imager (Fuji) with the Multi Gauge software (Fuji).

The *in vitro* D-loop extension assay was performed as described previously [27]. Briefly, primer extension was initiated by formation of D-loop with either Rad54 or Rad54-AA (see above) followed by incubation with RPA (660 nM), PCNA (2.5, 5, 10 and 20 nM), RFC (10 nM) and Pol δ (15 nM) in buffer O (20 mM Tris-Cl, pH 7.5, 5 mM DTT, 0.1 mM EDTA, 150 mM KCl, 40 μg/ml BSA, 8 mM MgCl2, 5% glycerol) and 100 μM each of dGTP and dCTP. The reaction mixtures were then incubated for 5 min at 30°C and DNA synthesis was initiated by addition of buffer S (100 μM dTTP and 0.375 μCi [α-^32^P] dATP in buffer O). After 10 min at 30°C, the reactions were stopped, deproteinized and loaded on a 0.8% (w/v) agarose gel. The gel was then dried on DE81 paper and exposed to phosphorimager screen and analyzed in Fuji FLA 9000 imager with the Multi Gauge software.

### In vivo primer extension assay

The *rad54-AA* and *rdh54-AA* alleles were introduced into the primer extension assay strain background [33] by transformation of the wild type assay strain using PCR-based allele replacement [60]. Primer sequences are shown in Supplementary information; all replacements were confirmed by DNA sequencing. *HO* induction, cell harvests, DNA extractions, and the primer extension assays were carried out as described in [65].

## Supporting Information

File S1 **Contains Figures S1–S6 and Tables S1–S2. Figure S1 Characterization of the Rad54-PCNA interaction. A. The Rad54 PIP-box mutant retains interaction with Rad51.** Rad51 was preincubated with Rad54 (lanes 1–4) or Rad54-AA (lanes 5–8) or alone (lanes 9–12) then mixed with Ni-NTA agarose beads. After washing, the bound proteins were eluted and the supernant (S), wash (W) and SDS eluate (E) fractions were analyzed on 12% SDS-PAGE. Input (I) lanes show starting material containing unbound protein as a control. **B. Rad51 outcompetes PCNA for interaction with Rad54.** In the pull-down experiment, Rad54 was either pre-incubated with Rad51 and then mixed with Affi-PCNA beads (lanes 2, 6), or first the complex between Rad54 and PCNA was formed, and later this complex was challenged with equimolar concentration of Rad51 (lanes 3, 7) or with 10 fold excess of Rad51 over PCNA (lanes 4, 8). In the control experiment, Rad54 was incubated with affi-PCNA beads (lanes 1, 5). Supernatant (S), and eluate (E) fractions were separated on a 12% SDS-PAGE gel, followed by Coomassie staining.**Figure S2 The Rad54 PCNA interaction mutant (AA) is defective in completion of recombination. A. Increased levels of Rad52 foci in the rad54Δ and** ***rad54-AA*** **mutants.** Shown are representative single Z-planes of wild type and *rad54-AA* strains expressing Rad52-RFP from the endogenous locus. Scale bar, 5 microns. **B. Rad52 foci last longer in the rad54Δ and** ***rad54-AA*** **mutants.** Points represent duration of individual foci, the line marks the mean duration for each strain. Significance from the wild type was determined by one-tailed T-test (p<0.05). **C.** ***rad54-AA*** **is synthetic lethal with** ***srs2Δ*** **.** Diploids heterozygous for *rad54-AA* and *srs2Δ* were sporulated and dissected. The phenotype of the non-viable spores were gleaned from that of viable sister spores. No viable *rad54-AA srs2Δ* were observed, while single mutants were observed at the predicted ratios. **D. Rad54-AA-YFP is expressed at similar levels to the Rad54-YFP protein and is able to be recruited to Rad52 recombination foci.** Shown is a representative Z-plane of cells expressing Rad52-RFP and either Rad54-YFP or Rad54-AA-YFP. Colocalization is shown in the RFP-YFP merge panel (RY merge) with orange arrows. Differential Interference Contrast (DIC) image is included to show cell morphology. Scale bar, 5 microns. **Figure S3** ***Rad54-AA*** **is defective in D-loop formation.** Rad51 (1 μM) was first nucleated on labeled ssDNA, followed by addition of increasing concentrations (75, 150, 300 nM) of Rad54 wild type (wt, lanes 2–4) or Rad54-AA (lanes 5–7), respectively. D-loop reactions were started by addition of the donor plasmid. Lane 1 represents control reaction with no Rad54. After the addition of gel loading buffer, the reaction mixtures were resolved in a 0.8% agarose gel in TAE buffer. **Figure S4** ***Rad54-AA*** **binds equally well short dsDNA oligonucleotide. A.** ***Rad54-AA*** **and wild type bind equally well to the short dsDNA in the absence of ATP.** Purified *S. cerevisiae* Rad54 and Rad54-AA (12.5, 25, 50, 100, 200 nM) were incubated for 10 min with fluorescently labelled dsDNA 49-mer in the absence of ATP. After the addition of gel loading buffer, the reaction mixtures were resolved in a 10% polyacrylamide gel in TBE buffer. **B. Rad54-AA and wild type bind equally well to the short dsDNA in the presence of ATP.** Purified *S. cerevisiae* Rad54 and Rad54-AA (12.5, 25, 50, 100, 200 nM) were incubated for 10 min with fluorescently labelled dsDNA 49-mer in the presence of 3 mM ATP. After the addition of gel loading buffer, the reaction mixtures were resolved in a 10% polyacrylamide gel in TBE buffer. **C. Quantification of the DNA binding reactions** shown in A and B. Error bars represent the standard error from three independent trials. **Figure S5 Rad54-L/Q binds dsDNA as efficiently as wild type Rad54. A. Rad54-L/Q proficiently binds DNA.** Purified *S. cerevisiae* Rad54 and Rad54-L/Q (31.25, 62.5, 125, 250, 500 or 1000 nM) were incubated for 10 min with linearized pBluescript plasmid to assess DNA binding. Prior to gel electrophoresis, the proteins were cross-linked to DNA with 0.1% glutaraldehyde. After the addition of gel loading buffer, the reaction mixtures were resolved in a 0.8% agarose gel in TAE buffer and stained with Midori Green DNA stain. **B. Quantification of the DNA binding reactions** shown in A. Error bars represent the standard error from three independent trials. **Figure S6 Rad54 PIP-box mutant analysis. A.** **PIP-box motif and the location of the new mutations.** The consensus sequence of the PIP-box and the amino acid sequence of Rad54 between Q488 and F495 is depicted. The mutations in Rad54 (Q488A; L491Q; F495H and L491Q,F495H) are shown below. **B. Rad54 F495H mutant retains wild type ATPase activity, while all others do not.** Rad54 wild type (wt), Rad54 Q488A (Rad54 Q/A), Rad54 L491Q (Rad54 L/Q), Rad54 F495H (Rad54-F/H) and Rad54 LF491,495QH (Rad54 LF/QH), respectively, were mixed with dsDNA and γ-[^32^P]-labeled ATP. At indicated times, samples were withdrawn and analyzed by thin-layer chromatography. Error bars represent standard error of 3 experiments. **C. Rad54 F/H is the only PIP-box mutant that is fully proficient in D-loop formation.** Reactions were performed as described in legend to Fig 4a The gels were quantified and plotted. Error bars represent standard error of 3 experiments. **D. Rad54 F/H complements the MMS sensitivity and Rad52 focus phenotype of** ***rad54Δ*** **cells.** Ten-fold serial dilutions of rad54Δ yeast cells, transformed with pTB326 (empty vector), pTB326-*RAD54*, and pTB326-*RAD54 F/H* plasmids, respectively, were spotted on selective media containing increasing concentration of MMS (0, 0.00125, 0.0025%). ++++ indicates four dilutions spots with detectable growth, + indicates that only the most concentrated spot exhibited growth. Relative levels of Rad52 foci are indicated with + as wild type levels, and ++ as a two-fold increase in spontaneous Rad52 foci.
(PDF)
