# Correction: The PCNA Interaction Protein Box Sequence in Rad54 Is an Integral Part of Its ATPase Domain and Is Required for Efficient DNA Repair and Recombination

**DOI:** 10.1371/journal.pone.0101095

**Published:** 2014-06-24

**Authors:** The PLOS ONE Staff

A reference is omitted from the fourth sentence of the second paragraph under the subheading “Mutational analysis of the PIP-box domain” in the Results and Discussion section.

The sentence should read: A complementary study performed independently in the Heyer laboratory came to the same set of conclusions (Zhang et al., 2013).

The reference is: Zhang X-P, Janke R, Kingsley J, Luo J, Fasching C, et al. (2013) A Conserved Sequence Extending Motif III of the Motor Domain in the Snf2-Family DNA Translocase Rad54 Is Critical for ATPase Activity. PLoS ONE 8(12): e82184. doi:10.1371/journal.pone.0082184
