# Supplementary material for: The PCNA Interaction Protein Box Sequence in Rad54 Is an Integral Part of Its ATPase Domain and Is Required for Efficient DNA Repair and Recombination
Source: PLoS One. 2013 Dec 20;8(12):e82630. doi: 10.1371/journal.pone.0082630 (PMC3869717; doi:10.1371/journal.pone.0082630)
Supplement: File S1 — Contains Figures S1–S6 and Tables S1–S2. Figure S1 Characterization of the Rad54-PCNA interaction. A. The Rad54 PIP-box mutant retains interaction with Rad51. Rad51 was preincubated with Rad54 (lanes 1–4) or Rad54-AA (lanes 5–8) or alone (lanes 9–12) then mixed with Ni-NTA agarose beads. After washing, the bound proteins were eluted and the supernant (S), wash (W) and SDS eluate (E) fractions were analyzed on 12% SDS-PAGE. Input (I) lanes show starting material containing unbound protein as a control. B. Rad51 outcompetes PCNA for interaction with Rad54. In the pull-down experiment, Rad54 was either pre-incubated with Rad51 and then mixed with Affi-PCNA beads (lanes 2, 6), or first the complex between Rad54 and PCNA was formed, and later this complex was challenged with equimolar concentration of Rad51 (lanes 3, 7) or with 10 fold excess of Rad51 over PCNA (lanes 4, 8). In the control experiment, Rad54 was incubated with affi-PCNA beads (lanes 1, 5). Supernatant (S), and eluate (E) fractions were separated on a 12% SDS-PAGE gel, followed by Coomassie staining.Figure S2 The Rad54 PCNA interaction mutant (AA) is defective in completion of recombination. A. Increased levels of Rad52 foci in the rad54Δ and rad54-AA mutants. Shown are representative single Z-planes of wild type and rad54-AA strains expressing Rad52-RFP from the endogenous locus. Scale bar, 5 microns. B. Rad52 foci last longer in the rad54Δ and rad54-AA mutants. Points represent duration of individual foci, the line marks the mean duration for each strain. Significance from the wild type was determined by one-tailed T-test (p<0.05). C. rad54-AA is synthetic lethal with srs2Δ . Diploids heterozygous for rad54-AA and srs2Δ were sporulated and dissected. The phenotype of the non-viable spores were gleaned from that of viable sister spores. No viable rad54-AA srs2Δ were observed, while single mutants were observed at the predicted ratios. D. Rad54-AA-YFP is expressed at similar levels to the Rad54-YFP protein [file pone.0082630.s001.pdf]

SUPPLEMENTARY INFORMATION

Table S1. Strains and Plasmids used in this study

Strain*	Genotype	Source
W5022-11D	*MAT***a** *RAD52-RFP MTW1-CFP RAD54-YFP*	This study
W5609-17C	*MAT***a** *RAD52-RFP MTW1-CFP rad54-Y494A,F495A-YFP*	This study
W5019-15C	*MAT***a** *RAD52-RFP MTW1-CFP RDH54-YFP RAD54*	This study
W5563-5B	*MAT***a** *RAD52-RFP MTW1-CFP RDH54-YFP rad54::LEU2*	This study
W5564-2C	*MAT***a** *RAD52-RFP MTW1-CFP RDH54-YFP rad54-Y494A F495A*	This study
W5762	*MAT***a**/α *trp1-1/TRP1 LYS2/lys2Δ srs2::HIS3/SRS2 rad54-Y494A F495A/RAD54*	This study
JKM161	*MAT***a** *HMLalpha Δhmr::ADE1 Δho ade1-100 ade3::GAL::HO leu2-3,112 lys5 trp1::hisG ura3*	N. Sugawara, J. Haber
tNS2045	*MAT***a** *HMLalpha Δhmr::ADE1 Δho ade1-100 ade3::GAL::HO leu2-3,112 lys5 trp1::hisG ura3 rad54::URA3*	N. Sugawara, J. Haber
U2829	*MAT***a** *HMLalpha Δhmr::ADE1 Δho ade1-100 ade3::GAL::HO leu2-3,112 lys5 trp1::hisG ura3 rdh54::KAN-MX*	This study
J1603	*MAT***a** *HMLalpha Δhmr::ADE1 Δho ade1-100 ade3::GAL::HO leu2-3,112 lys5 trp1::hisG ura3 rdh54-F511A,F512A*	This study
J1604	*MAT***a** *HMLalpha Δhmr::ADE1 Δho ade1-100 ade3::GAL::HO leu2-3,112 lys5 trp1::hisG ura3 rad54-F494A, Y495A*	This study
J1607	*MAT***a** *HMLalpha Δhmr::ADE1 Δho ade1-100 ade3::GAL::HO leu2-3,112 lys5 trp1::hisG ura3 rad54-F494A, Y495A rdh54::KAN-MX*	This study
J1608	*MAT***a** *HMLalpha Δhmr::ADE1 Δho ade1-100 ade3::GAL::HO leu2-3,112 lys5 trp1::hisG ura3 rad54-F494A, Y495A rdh54-F511A,F512A*	This study
HKY950-12C	*MAT*α *leu2-3,112 his3-11,15 ADE2 ura3-1 trp1-1 CAN1 RAD5*	This study
HKY1026-1B	*MAT***a** *leu2-3,112 his3-11,15 ade2-1 ura3-1 trp1-1 can1-100 hom3-10 RAD5*	This study
HKY953-3A	*MAT***a** *leu2-3,112 his3-11,15 ADE2 ura3-1 trp1-1 CAN1 RAD5*	This study
HKY947-14D	*MAT*α *leu2-3,112 his3-11,15 ade2-1 ura3-1 trp1-1 can1-100 hom3-10 RAD5*	This study

HKY950-15B	*MAT***a** *rad54::HIS3 leu2-3,112 his3-11,15 ADE2 ura3-1 trp1-1 CAN1 RAD5*	This study
HKY947-22C	*MATα rad54::HIS3 leu2-3,112 his3-11,15 ade2-1 ura3-1 trp1-1 can1-100 hom3-10 RAD5*	This study
HKY947-9D	*MATα rad54::HIS3 leu2-3,112 his3-11,15 ade2-1 ura3-1 trp1-1 can1-100 hom3-10 RAD5*	This study
MSY144-2A	*MAT***a** *rad54-Y494A F495A leu2-3,112 his3-11,15 ade2-1 ura3-1 trp1-1 can1-100 hom3-10 RAD5*	This study
MSY145-3D	*MATα rad54-Y494A F495A leu2-3,112 his3-11,15 ADE2 ura3-1 trp1-1 CAN1 RAD5*	This study
MSY144-3A	*MATα rad54-Y494A F495A leu2-3,112 his3-11,15 ade2-1 ura3-1 trp1-1 can1-100 hom3-10 RAD5*	This study
MSY145-2C	*MAT***a** *rad54-Y494A F495A leu2-3,112 his3-11,15 ADE2 ura3-1 trp1-1 CAN1 RAD5*	This study
MSY41-3C	*MATα leu2-ecori::URA3-leu2-bsteii his3-11,15 ade2-1 ura3-1 trp1-1 can1-100 RAD5*	This study
MSY41-2D	*MATα leu2-ecori::URA3-leu2-bsteii his3-11,15 ade2-1 ura3-1 trp1-1 can1-100 RAD5*	This study
MSY41-5A	*MATα leu2-ecori::URA3-leu2-bsteii his3-11,15 ade2-1 ura3-1 trp1-1 can1-100 RAD5*	This study
HKY813-2D	*MATα rad54::HIS3 leu2-ecori::URA3-leu2-bsteii his3-11,15 ade2-1 ura3-1 trp1-1 can1-100 RAD5*	This study
HKY813-9B	*MAT***a** *rad54::HIS3 leu2-ecori::URA3-leu2-bsteii his3-11,15 ade2-1 ura3-1 trp1-1 can1-100 RAD5*	This study
HKY813-10A	*MAT***a** *rad54::HIS3 leu2-ecori::URA3-leu2-bsteii his3-11,15 ade2-1 ura3-1 trp1-1 can1-100 RAD5*	This study
MSY142-1B	*MATα rad54-Y494A F495A leu2-ecori::URA3-leu2-bsteii his3-11,15 ade2-1 ura3-1 trp1-1 can1-100 RAD5*	This study
MSY142-2A	*MATα rad54-Y494A F495A leu2-ecori::URA3-leu2-bsteii his3-11,15 ade2-1 ura3-1 trp1-1 can1-100 RAD5*	This study
MSY142-7C	*MATα rad54-Y494A F495A leu2-ecori::URA3-leu2-bsteii his3-11,15 ade2-1 ura3-1 trp1-1 can1-100 RAD5*	This study
HKY1025-47D	*MATα leu2-3,112 his3-11,15 ADE2 ura3-1 trp1-1 CAN1 RAD5*	This study
HKY1105-3A	*MATα rad54::LEU2 leu2-3,112 his3-11,15 ADE2 ura3-1 trp1-1 CAN1 RAD5*	This study

*All strains in this study are isogenic to W3749-1A [1], an *ADE2 bar1::LEU2 RAD5* derivative of W303-1A (*MAT***a** *ade2-1 can1-100 ura3-1 his3-11,15 leu2-3,112 trp1-1 rad5-535*) [2] unless otherwise specified in the genotype.

Table S2. Oligonucleotides used in the study

Name	Sequence	Purpose
D1 oligonucleotide	5'AAATCAATCTAAAGTATATATGAGTAAACTTGGTCTGACAGTTACCAATGCTTAATCAGTGAGGCACCTATCTCAGCGATCTGTCTATTT3'	D-loop assay
mobHJ oligo1	5'GACGCTGCCGAATTCTACCAGTGCCTTGCTAGGACATCTTTGCCCACCTGCAGGTTCACCC3'	Branch-migration assay
mobHJ oligo2	5'TGGGTGAACCTGCAGGTGGGCAAAGATGTCCATCTGTTGTAATCGTCAAGCTTTATGCCGTT3'	Branch-migration assay
mobHJ oligo3	5'GGGTGAACCTGCAGGTGGGCAAAAATGTCCTAGCAAGGCACTGGTAGAATTCGGCAGCGTC3'	Branch-migration assay
mobHJ oligo4	5'GAACGGCATAAAGCTTGACGATTACAACAGATGGACATTTTTGCCCACCTGCAGGTTCACCC3'	Branch-migration assay
dsDNA oligo1	5'AGCTACCATGCCTGCACGAATTAAGCAATTCGTAATCATGGTCATAGCT3'	EMSA
dsDNA oligo2	5'AGCTATGACCATGATTACGAATTGCTTAATTCGTGCAGGCATGGTAGCT3'	EMSA

Supplementary methods

Oligo-based DNA Mobility Shift Assay

Purified *S. cerevisiae* Rad54 and Rad54-AA (12.5, 25, 50, 100 or 200 nM) were incubated with fluorescently labeled dsDNA 49-mer (3 nM) at 30°C in 10 µl of buffer D (40 mM Tris-HCl, pH 7.8, 50 mM KCl, 1 mM dithiothreitol, and 100 µg/ml bovine serum albumin) for 10 min. Where indicated, 3 mM ATP was present in the reaction. After the addition of gel loading buffer, the reaction mixtures were resolved in 10% native polyacrylamide gels in TAE buffer at 4°C. The fluorescent DNA species were visualized and quantified in the Fuji FLA 9000 imager with the Multi Gauge software (Fuji).

Supplementary figure legends

Supplementary Figure 1. Characterization of the Rad54-PCNA interaction.

A. The Rad54 PIP-box mutant retains interaction with Rad51. Rad51 was preincubated with Rad54 (lanes 1-4) or Rad54-AA (lanes 5-8) or alone (lanes 9-12) then mixed with Ni-NTA agarose beads. After washing, the bound proteins were eluted and the supernant (S), wash (W) and SDS eluate (E) fractions were analyzed on 12% SDS-PAGE. Input (I) lanes show starting material containing unbound protein as a control.

B. Rad51 outcompetes PCNA for interaction with Rad54. In the pull-down experiment, Rad54 was either pre-incubated with Rad51 and then mixed with Affi-PCNA beads (lanes 2, 6), or first the complex between Rad54 and PCNA was formed, and later this complex was challenged with equimolar concentration of Rad51 (lanes 3, 7) or with 10 fold excess of Rad51 over PCNA (lanes 4, 8). In the control experiment, Rad54 was incubated with affi-PCNA beads (lanes 1, 5). Supernatant (S), and eluate (E) fractions were separated on a 12% SDS-PAGE gel, followed by Coomassie staining.

Supplementary Figure 2. The Rad54 PCNA interaction mutant (AA) is defective in completion of recombination.

A. Increased levels of Rad52 foci in the *rad54Δ* and *rad54-AA* mutants. Shown are representative single Z-planes of wild type and *rad54-AA* strains expressing Rad52-RFP from the endogenous locus. Scale bar, 5 microns.

B. Rad52 foci last longer in the *rad54*Δ and *rad54-AA* mutants. Points represent duration of individual foci, the line marks the mean duration for each strain. Significance from the wild type was determined by one-tailed T-test ($p < 0.05$).

C. *rad54-AA* is synthetic lethal with *srs2*Δ. Diploids heterozygous for *rad54-AA* and *srs2*Δ were sporulated and dissected. The phenotype of the non-viable spores were gleaned from that of viable

sister spores. No viable *rad54-AA srs2Δ* were observed, while single mutants were observed at the predicted ratios.

D. Rad54-AA-YFP is expressed at similar levels to the Rad54-YFP protein and is able to be recruited to Rad52 recombination foci. Shown is a representative Z-plane of cells expressing Rad52-RFP and either Rad54-YFP or Rad54-AA-YFP. Colocalization is shown in the RFP-YFP merge panel (RY merge) with orange arrows. Differential Interference Contrast (DIC) image is included to show cell morphology. Scale bar, 5 microns.

Supplementary Figure 3. Rad54-AA is defective in D-loop formation.

Rad51 (1 μM) was first nucleated on labeled ssDNA, followed by addition of increasing concentrations (75, 150, 300 nM) of Rad54 wild type (wt, lanes 2-4) or Rad54-AA (lanes 5-7), respectively. D-loop reactions were started by addition of the donor plasmid. Lane 1 represents control reaction with no Rad54. After the addition of gel loading buffer, the reaction mixtures were resolved in a 0.8% agarose gel in TAE buffer.

Supplementary Figure 4. Rad54-AA binds equally well short dsDNA oligonucleotide.

A. Rad54-AA and wild type bind equally well to the short dsDNA in the absence of ATP. Purified *S. cerevisiae* Rad54 and Rad54-AA (12.5, 25, 50, 100, 200 nM) were incubated for 10 min with fluorescently labelled dsDNA 49-mer in the absence of ATP. After the addition of gel loading buffer, the reaction mixtures were resolved in a 10% polyacrylamide gel in TBE buffer.

B. Rad54-AA and wild type bind equally well to the short dsDNA in the presence of ATP. Purified *S. cerevisiae* Rad54 and Rad54-AA (12.5, 25, 50, 100, 200 nM) were incubated for 10 min with fluorescently labelled dsDNA 49-mer in the presence of 3 mM ATP. After the addition of gel loading buffer, the reaction mixtures were resolved in a 10% polyacrylamide gel in TBE buffer.

C. Quantification of the DNA binding reactions shown in A and B. Error bars represent the standard error from three independent trials.

Supplementary Figure 5. Rad54-L/Q binds dsDNA as efficiently as wild type Rad54.

A. Rad54-L/Q proficiently binds DNA. Purified *S. cerevisiae* Rad54 and Rad54-L/Q (31.25, 62.5, 125, 250, 500 or 1000 nM) were incubated for 10 min with linearized pBluescript plasmid to assess DNA binding. Prior to gel electrophoresis, the proteins were cross-linked to DNA with 0.1% glutaraldehyde. After the addition of gel loading buffer, the reaction mixtures were resolved in a 0.8 % agarose gel in TAE buffer and stained with Midori Green DNA stain.

B. Quantification of the DNA binding reactions shown in A. Error bars represent the standard error from three independent trials.

Supplementary Figure 6. Rad54 PIP-box mutant analysis.

A. PIP-box motif and the location of the new mutations. The consensus sequence of the PIP-box and the amino acid sequence of Rad54 between Q488 and F495 is depicted. The mutations in Rad54 (Q488A; L491Q; F495H and L491Q,F495H) are shown below.

B. Rad54 F495H mutant retains wild type ATPase activity, while all others do not. Rad54 wild type (wt), Rad54 Q488A (Rad54 Q/A), Rad54 L491Q (Rad54 L/Q), Rad54 F495H (Rad54-F/H) and Rad54 LF491,495QH (Rad54 LF/QH), respectively, were mixed with dsDNA and γ-[^{32}P]-labeled ATP. At indicated times, samples were withdrawn and analyzed by thin-layer chromatography. Error bars represent standard error of 3 experiments.

C. Rad54 F/H is the only PIP-box mutant that is fully proficient in D-loop formation. Reactions were performed as described in legend to Fig 4a The gels were quantified and plotted. Error bars represent standard error of 3 experiments.

D. Rad54 F/H complements the MMS sensitivity and Rad52 focus phenotype of *rad54Δ* cells. Ten-fold serial dilutions of *rad54Δ* yeast cells, transformed with pTB326 (empty vector), pTB326-*RAD54*, and pTB326-*RAD54 F/H* plasmids, respectively, were spotted on selective media containing increasing concentration of MMS (0, 0.00125, 0.0025%). ++++ indicates four dilutions spots with detectable growth, + indicates that only the most concentrated spot exhibited growth. Relative levels of Rad52 foci are indicated with + as wild type levels, and ++ as a two-fold increase in spontaneous Rad52 foci.

Supplementary references:

1. Lisby M, Barlow JH, Burgess RC, Rothstein R (2004) Choreography of the DNA damage response: spatiotemporal relationships among checkpoint and repair proteins. Cell 118: 699-713.
2. Thomas BJ, Rothstein R (1989) Elevated recombination rates in transcriptionally active DNA. Cell 56: 619-630.

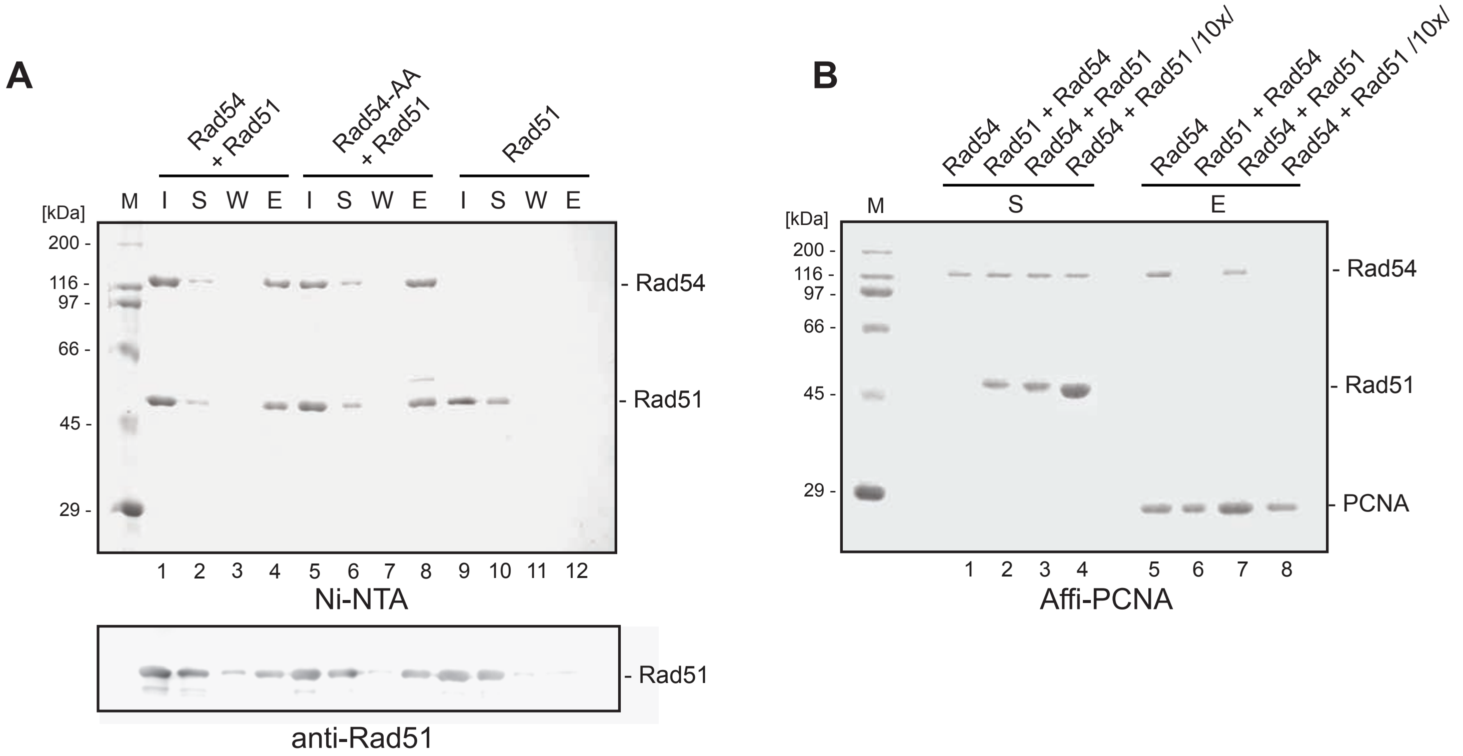

Supplementary figure 1

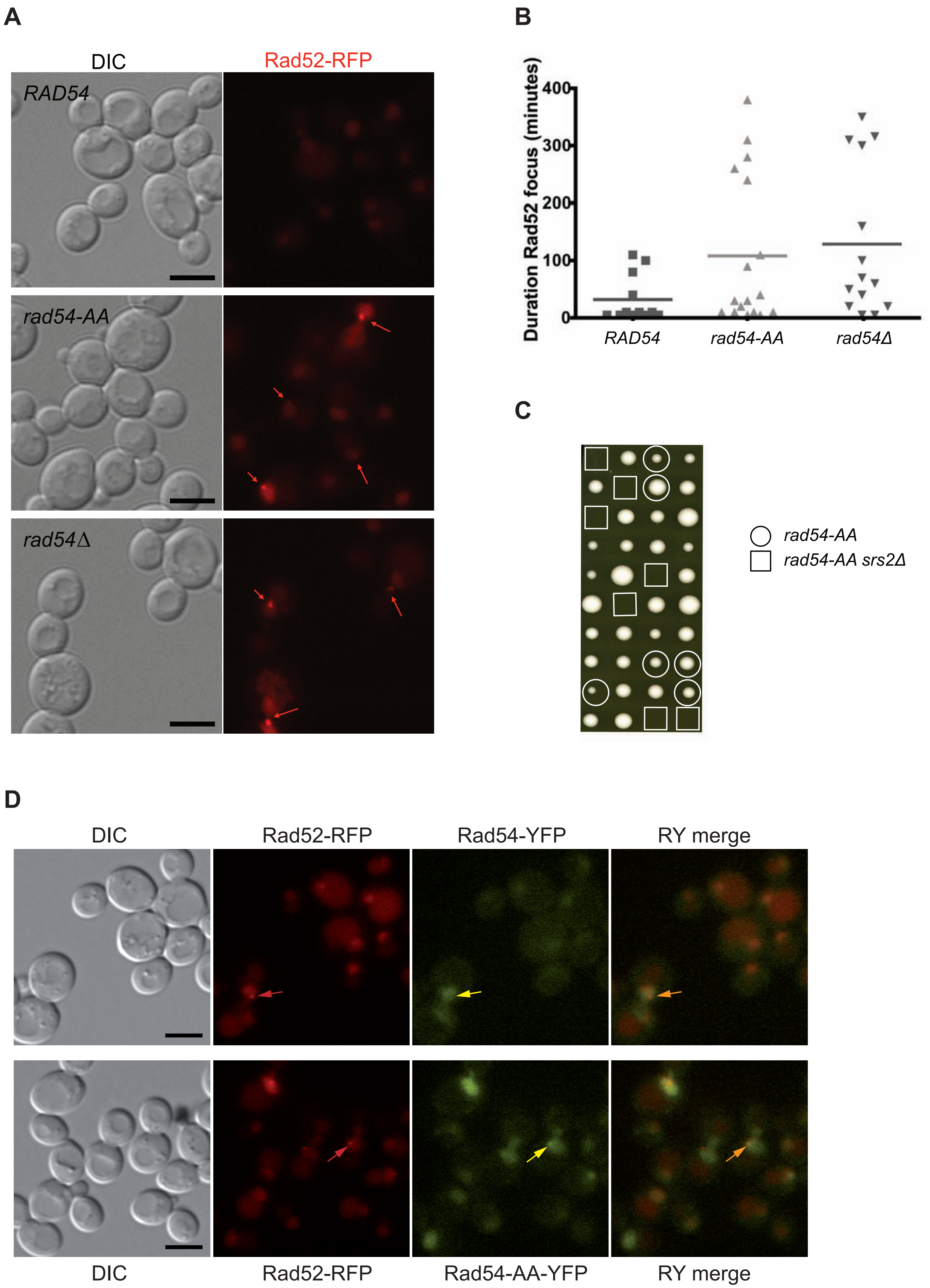

Supplementary figure 2

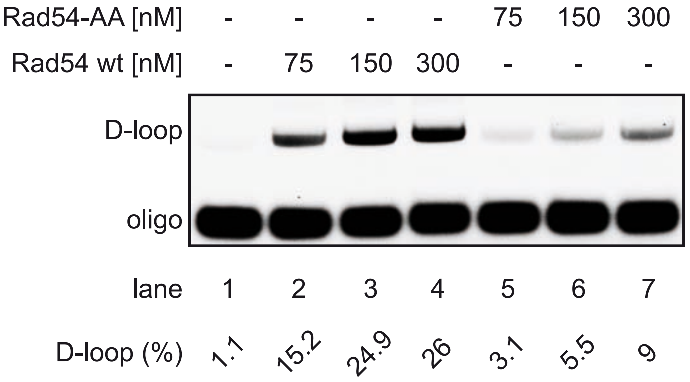

Supplementary figure 3

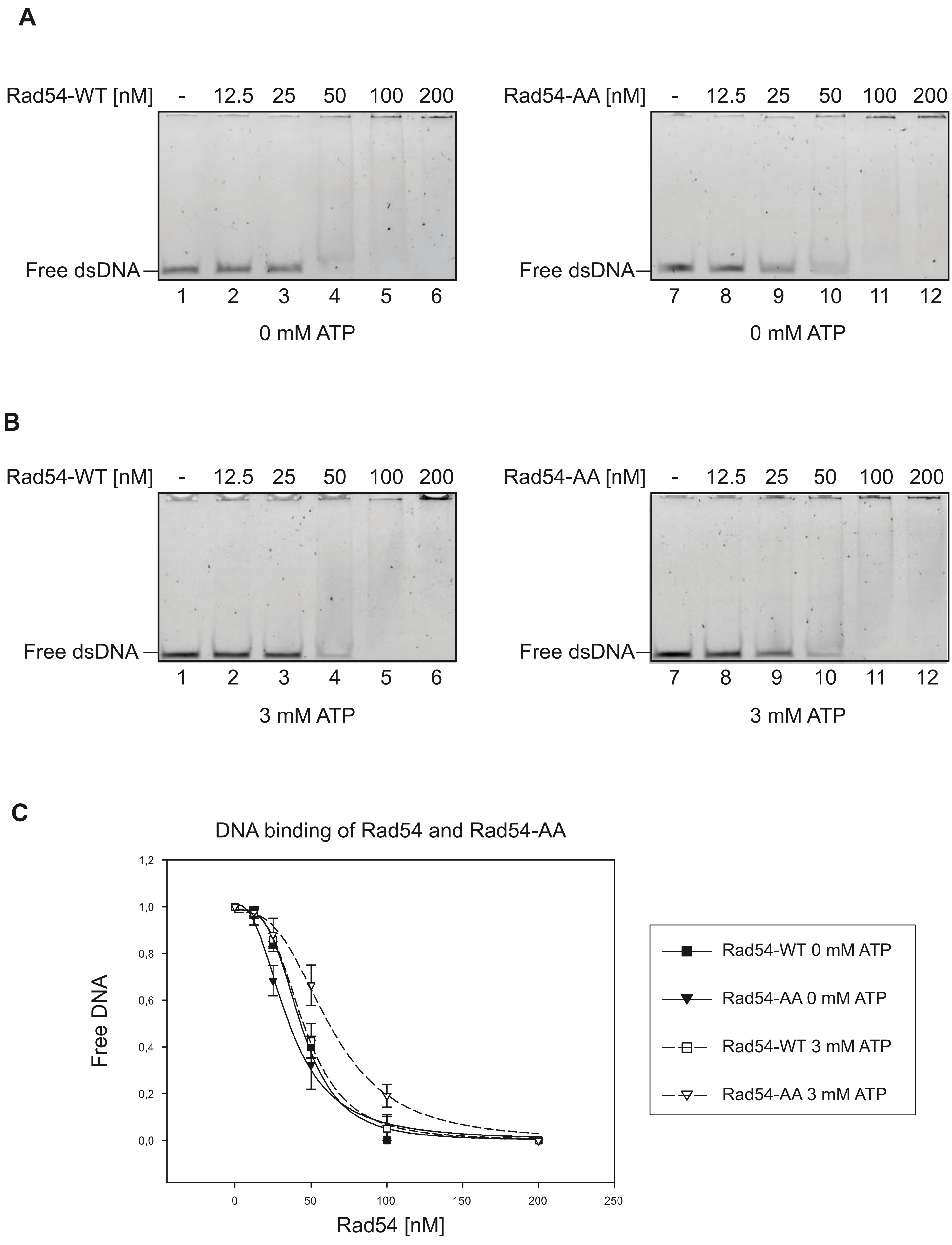

Supplementary Figure 4

A

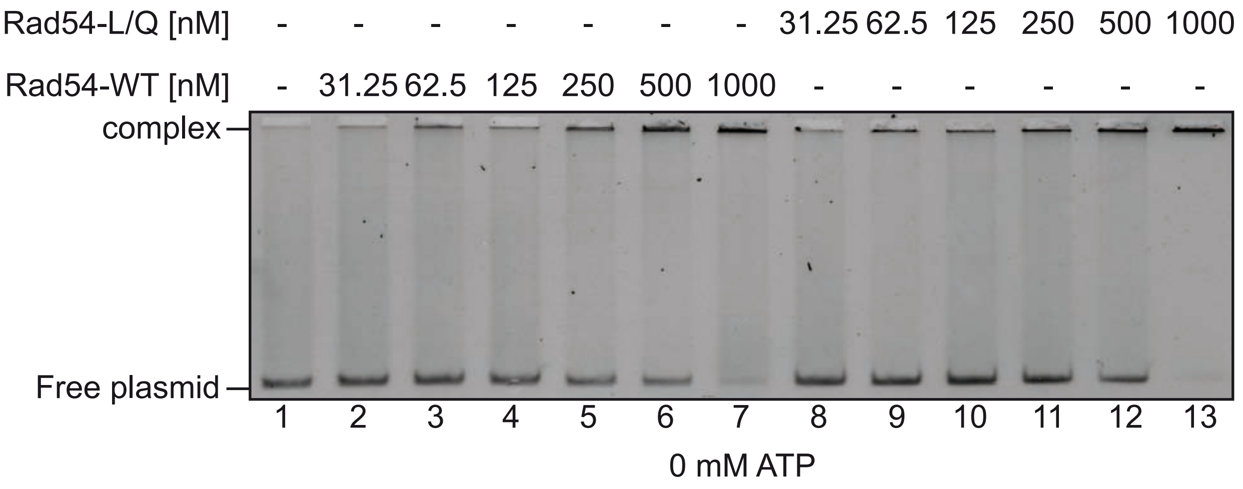

B

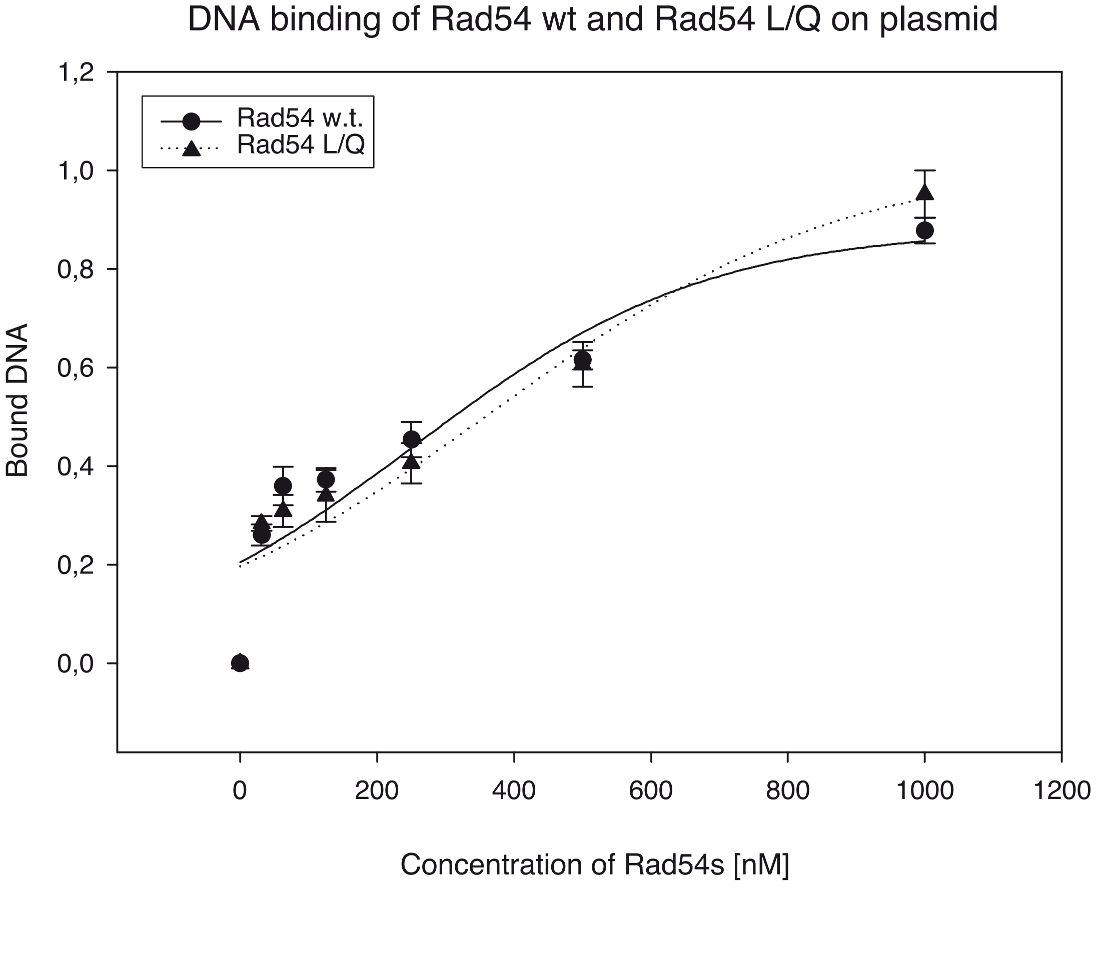

Supplementary figure 5

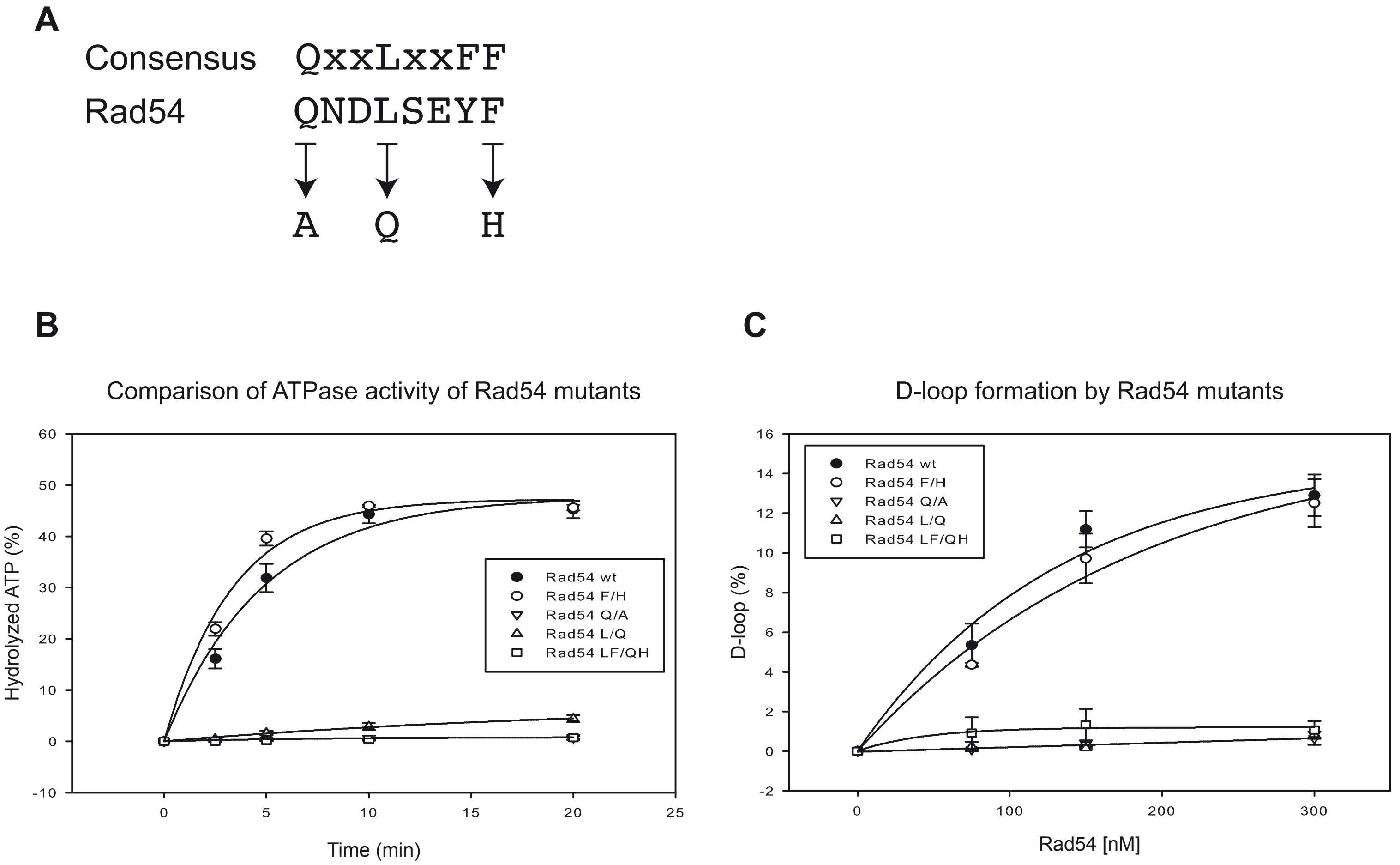

D

	MMS resistance	Spontaneous Rad52 foci
RAD54	++++	+
rad54-FH	++++	+
rad54Δ	+	++

Supplementary figure 6
